# Synchrotron total-scattering data applicable to dual-space structural analysis

**DOI:** 10.1107/S2052252521001664

**Published:** 2021-03-06

**Authors:** Jonas Beyer, Kenichi Kato, Bo Brummerstedt Iversen

**Affiliations:** a Department of Chemistry, Aarhus University, Langelandsgade 140, Aarhus C, 8000, Denmark; b RIKEN SPring-8 Center, 1-1-1 Kouto, Sayo-cho, Sayo-gun, Hyogo, 679-5148, Japan; c JST, PRESTO, 4-1-8 Honcho, Kawaguchi, Saitama 332-0012, Japan

**Keywords:** dual-space structural analysis, total-scattering data, synchrotrons, pair distribution functions, powder X-ray diffraction

## Abstract

Synchrotron powder X-ray diffraction (PXRD) is a well established technique for investigating the atomic arrangement of crystalline materials. At modern beamlines, X-ray scattering data can be collected in a total-scattering setting, which additionally opens up the opportunity for direct-space structural analysis through the atomic pair distribution function (PDF). Modelling of PXRD and PDF data is typically carried out separately, but employing a concurrent structural model to both direct- and reciprocal-space data has the possibility to enhance total-scattering data analysis. However, total-scattering measurements applicable to such dual-space analyses are technically demanding. Recently, the technical demands have been fulfilled by a MYTHEN microstrip detector system (OHGI), which meets the stringent requirements for both techniques with respect to *Q* range, *Q* resolution and dynamic range. In the present study, we evaluate the quality of total-scattering data obtained with OHGI by separate direct- and reciprocal-space analysis of Si. Excellent agreement between structural parameters in both spaces is found, demonstrating that the total-scattering data from OHGI can be utilized in dual-space structural analysis *e.g.* for *in situ* and *operando* measurements.

## Abstract 2

Synchrotron X-ray scattering data with high *Q* resolution, *Q* range and dynamic range are collected using the OHGI detector system, and excellent correspondence is achieved in structural refinements of the data in reciprocal and direct space.

## 1. Introduction   

The study of the solid phase of matter has improved significantly in recent decades causing immense progress in fields such as life science and materials science. The progress can be mainly attributed to two aspects. One is the increasing number of large science facilities such as spallation neutron sources and synchrotrons, which offer users access to increasingly bright and brilliant neutrons and X-rays. The other is a steady improvement in detector technology leading to short acquisition times and high data quality. The enhanced quality of data is a call for increasingly sophisticated analysis techniques, which can give detailed structural descriptions of the solid phase in terms of chemical bonding, microstructure and lattice defects.

Powder X-ray diffraction (PXRD) is a well established technique in the materials science community for determining phase purity, composition, microstructural features and the crystallographic structure of a powdered sample. Previous studies have shown that the accuracy of PXRD data collected is comparable with that of single-crystal X-ray diffraction (SCXRD) data in the case of crystalline materials with high symmetry (Tolborg *et al.*, 2017 ▸; Svane *et al.*, 2019 ▸).

For a perfect crystal, only the Bragg scattering from PXRD or SCXRD is needed to describe the crystalline structure. However, almost all crystalline materials exhibit disorder to some extent such as vacancies, dislocations, stacking faults or more complicated deviations from the average structure, such as incommensurable substructures or correlated thermal motion. Disorder and deviations from the average structure lead to diffuse scattering.

In a total-scattering experiment, Bragg and diffuse scattering are measured impartially. Analysis of total-scattering data can be carried out in reciprocal space using the Debye scattering equation. This technique *e.g.* has been used to model planar defects, morphology and correlated thermal motion of nanocrystals with poorly defined Bragg scattering (Scardi & Gelisio, 2016 ▸; Moscheni *et al.*, 2018 ▸; Bertolotti *et al.*, 2020 ▸). Total-scattering data can also be treated in direct space via a Fourier transformation to obtain the pair distribution function (PDF) (Egami & Billinge, 2012 ▸). For crystalline structures with well defined Bragg scattering and minor degrees of diffuse scattering from short-range disorder, modelling of the PDF is the method of choice (Scardi & Gelisio, 2016 ▸). Both techniques have also been successful for modelling of liquid and amorphous phases.

Considering the obvious scientific motivation, only a few total-scattering beamlines have been developed at synchrotrons for simultaneous, *i.e.* single shot, measurement of high-quality Bragg and diffuse scattering, referred to herein as dual-space quality. The primary reason is the inherent trade-off between range and resolution of the scattering vector, *Q*. Typical PXRD-dedicated beamlines have focused on high angular-resolution measurement of Bragg scattering with point detectors and relatively low energy X-rays (Fitch, 2004 ▸), while the focus of PDF dedicated beamlines has been on wide *Q*-range measurements with large area detectors and high-energy X-rays (Chupas *et al.*, 2007 ▸). Consequently, the average structure analysis, *e.g.* using the Rietveld method (Rietveld, 1969 ▸; Young, 2002 ▸), has been performed separately from local structural analysis using PDF methods. This situation makes it complicated to directly compare the local and average structure in crystalline solids with disorder using X-rays.

Accordingly, there is an increasing need for dual-space-quality total X-ray scattering data that permit average- and local-structure analysis on an equal basis, similar to the progress achieved for neutron time-of-flight diffractometers (Bowron *et al.*, 2010 ▸; Neuefeind *et al.*, 2012 ▸; Smith *et al.*, 2019 ▸). Dual-space-quality total-scattering data have been utilized to study *e.g.* oxygen disorder in δ-Bi_2_O_3_ (Hull *et al.*, 2009 ▸), where ‘big box’ modelling was carried out with the *RMCProfile* software (Tucker *et al.*, 2007 ▸).

Dual-space analysis requires the measurement of total-scattering data with a high dynamic range and high signal-to-noise ratio in order to be sensitive to Bragg and diffuse scattering. Furthermore, both the *Q* range and *Q* resolution need to be high in order to obtain well resolved diffraction peaks to a high order. The microstrip detector module MYTHEN (DECTRIS) (Schmitt *et al.*, 2003 ▸) has the potential for dual-space-quality total-scattering measurements because of a flexible arrangement to cover a wide *Q* range, high spatial resolution given by a sharp line-spread function and a photon-counting architecture with high signal-to-noise ratio. Even so, data obtained through MYTHEN modules have not been successfully applied to dual-space analysis. The unsuccessful attempts can be ascribed to the difference in X-ray response between microstrip channels, which is referred to as X-ray response non-uniformity (XRNU) (Kato *et al.*, 2019 ▸). XRNU is a major contributing factor in the dynamic range of a detector system. Although MYTHEN has a counter of 24 bits, which is equivalent to a dynamic range of 10^7^, a noise level of 1% caused by XRNU reduces the effective dynamic range to 10^4^. All types of X-ray detectors have been reported to suffer from XRNU (Amemiya, 1995 ▸; Williams & Shaddix, 2007 ▸; Bergamaschi *et al.*, 2010 ▸; Skinner *et al.*, 2012 ▸; Wernecke *et al.*, 2014 ▸). The conventional approach to the problem, the so-called flat-field calibration (Hammersley *et al.*, 1995 ▸; Moy *et al.*, 1996 ▸), succeeded in reducing the XRNU noise level from several percent down to 1%. However, the flat-field calibration, which needs a uniform reference intensity, has failed to reduce the level further because it is impossible to produce a completely uniform intensity. Recently, Kato *et al.* (Kato *et al.*, 2019 ▸; Kato & Shigeta, 2020 ▸) have developed a data-driven approach to the problem without using a uniform intensity, which is referred to as ReLiEf (response-to-light effector). The ReLiEf approach has succeeded in reducing the XRNU noise level in MYTHEN modules down to 0.1%, which is equivalent to a dynamic range of 10^6^. The ReLiEf algorithm has been integrated into a total-scattering measurement system called OHGI (overlapped high-grade intelligencer) (Kato *et al.*, 2019 ▸), which consists of fifteen overlapping MYTHEN modules installed at the RIKEN Materials Science beamline BL44B2 (Kato *et al.*, 2010 ▸; Kato & Tanaka, 2016 ▸) at SPring-8. A unique combination of OHGI, ReLiEf and intermediate energy (30 keV) X-rays facilitate the collection of single-shot dual-space-quality total X-ray scattering data with a wide *Q* range (*Q*_max_ > 25 Å^−1^), high *Q* resolution (*Q* step < 10^−3^ Å^−1^) and high signal-to-noise ratio (dynamic range > 10^5^). The precision in the intensity of the instrument has been shown to closely follow the Poisson distribution, even for weak signals, and the pristine data quality has been demonstrated for amorphous SiO_2_ and TiO_2_ nanoparticles (Kato *et al.*, 2019 ▸).

In the present study, we evaluate total-scattering data obtained through OHGI by analysis of a NIST standard reference material Si powder. Si is a highly crystalline material with minimal disorder and therefore serves as an appropriate reference material for a benchmark test. The structural features of Si have been fully understood from the viewpoint of both theory and experiment. One feature of the OHGI total-scattering data is outstanding reciprocal-space range and resolution, which allows computation of long-range PDFs (*r* > 500 Å) from a single-shot measurement. The primary purpose is to confirm consistency between structural parameters, such as lattice parameters and atomic displacement parameters (ADPs) obtained separately by Rietveld and PDF analysis. Moreover, the instrumental effects on the extremely well resolved PDFs are assessed by a boxcar-refinement scheme.

## 2. Methods   

### 2.1. Total-scattering measurements   

Total-scattering data of an Si powder (NIST SRM640d) were collected at 100 and 300 K using OHGI (Kato *et al.*, 2019 ▸) at the RIKEN Materials Science beamline BL44B2 (Kato *et al.*, 2010 ▸; Kato & Tanaka, 2016 ▸) at SPring-8. The incident X-rays had an energy of 27.546 (6) keV (λ = 0.4501 (1) Å), which was calibrated through Le Bail refinement (Le Bail, 2005 ▸) of LaB_6_ powder (NIST SRM660b) data. This corresponds to a *Q*_max_ of ∼27 Å^−1^. The detectable energy threshold of OHGI was set to 13.8 keV, which is equivalent to half of the incident energy, to minimize the effect of double photon counting. A single data set from OHGI has a 2θ step of 0.01°. In the present experiments, two data sets were collected by shifting OHGI 0.005° along 2θ. These were then integrated into a single data set with an effective resolution of 0.005°. The Si sample was packed into a glass capillary with an inner diameter of 0.3 mm. In addition, data were measured on an empty capillary at 100 and 300 K to subtract the background and instrument scattering before data normalization. By using an incident energy of 27.546 (6) keV and a capillary diameter of 0.3 mm, the absorption effect on the scattering intensity was negligible. Data processing was based on the assumption that the incident beam from the bending magnet X-ray source was completely polarized in the horizontal plane. The dimensions of the incident beam were fixed by a collimator 3 mm in the horizontal direction and 0.5 mm in the vertical direction. The total data-collection time at each temperature was ∼1 h.

To compare instrumental profile resolutions with beamlines that have *in situ* or *operando* capabilities, LaB_6_ powder data collected at two other synchrotron beamlines, P02.1 (Dippel *et al.*, 2015 ▸) and P21.1, at PETRA III (DESY in Hamburg, Germany) were analyzed. LaB_6_ powder data collected using an imaging plate (IP) detector at BL44B2 at SPring-8 have also been used to compare the instrumental profile resolutions.

### 2.2. Reciprocal-space refinements   

Rietveld and Le Bail refinements of the total-scattering data were carried out using the *TOPAS-Academic* Version 6 software (Coelho, 2018 ▸). An angular range from 6 to 110° 2θ was selected for data analysis. The background scattering was fitted using a seven-degree Chebyshev polynomial. Bragg peak profiles were modelled using the Thompson–Cox–Hastings (TCH) pseudo-Voigt peak-profile function (Thompson *et al.*, 1987 ▸). The number of peak-profile parameters was minimized by iteratively assessing the correlation matrix and *R* factors. Peak shifts and peak asymmetry, caused by misaligned sample capillaries and axial divergence, respectively, were insignificant. Two *R* factors, *R*_wp_ and *R*_Bragg_, were used to assess the reliability of fit; the former for evaluating the statistical significance of each data point, the latter for evaluating the difference between model and data at the calculated peak positions.

For the Le Bail refinements of LaB_6_, the refined parameters were a scale factor, background parameters, incident-beam wavelength and peak-profile parameters. The lattice parameter was fixed to the certified value of SRM660b (Black *et al.*, 2011 ▸). The refinement range was limited to 80° in 2θ owing to restrictions on the number of refinement parameters in *TOPAS*. For the Rietveld refinements of Si (space group #227 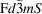, origin choice 2), the following parameters were employed: a scale factor, background parameters, the lattice parameter, TCH peak-profile parameters (*U*, *W* and *Y*) and the isotropic ADP. To avoid local minima, the ADPs were optimized by refining the model with 10 000 iterations, where a random number between −50 and 50% of the ADP after a convergent iteration was added to the value for the next iteration. The ADP with the lowest *R*_wp_ among all convergent iterations was selected as the final value.

### 2.3. Direct-space refinements   

PDFs of Si were computed using the *PDFgetX3* algorithm (Juhás *et al.*, 2013 ▸). The *PDFgetX3* data normalization is semi-quantative and not strictly correct for an arbitrary system. However, in the case of a homoatomic sample, such as Si, the normalization will be correct. The *Q* range was 1.0–27.0 Å^−1^ and the *ad hoc* correction parameter *r*_poly_ was set to 1.05. Least-squares refinements of the PDF model were also carried out using *TOPAS-Academic* Version 6, which allowed for refinements of long-range PDFs within reasonable time frames (Coelho *et al.*, 2015 ▸; Coelho, 2018 ▸). Refinements were carried out in a range of 1.0–500.0 Å with a step of 0.01 Å, with all points included. A convoluted sinc function was implemented to account for Fourier ripples (Chung & Thorpe, 1997 ▸). Refinement parameters for Si were as follows: a scale factor, the lattice parameter, the isotropic ADP, and instrumental parameters *Q*_damp_ and *Q*_broad_. The *R*_wp_ value was used to assess the fit. To avoid local minima, the ADPs were optimized by refining the model with 1000 iterations, where a random number between −25 and 25% of the ADP after a convergent iteration was added to the value for the next iteration. The ADP with the lowest *R*_wp_ among all convergent iterations was selected as the final value.

### 2.4. Boxcar refinements in direct space   

To examine the behaviour of the PDF as a function of correlation length *r*, a boxcar-refinement scheme was employed (Proffen & Kim, 2009 ▸; Usher *et al.*, 2016 ▸). A narrow section of the direct-space range, *i.e.* a box, was defined and subsequently moved in fixed steps through the entire range. The box width was set to 10 Å and the centroid was moved in 10 Å steps in the range 1–500 Å. Note that the first box had a width of 9 Å. At first, three refinement parameters were included in the model; scale factor, the isotropic ADP and the lattice parameter. The lattice parameter was found to be consistent between all ranges and was therefore fixed at the value found from Rietveld refinements. A convoluted sinc function was included to account for the Fourier ripples. The final refinement parameters were a scale factor and the isotropic ADP. Parameters in each box were refined with 100 iterations. Similar to the description in Section 2.3, the final ADP was optimized by using a random number between −50 and 50% after each convergent iteration.

## 3. Quality of total-scattering data   

**Figure 1:**
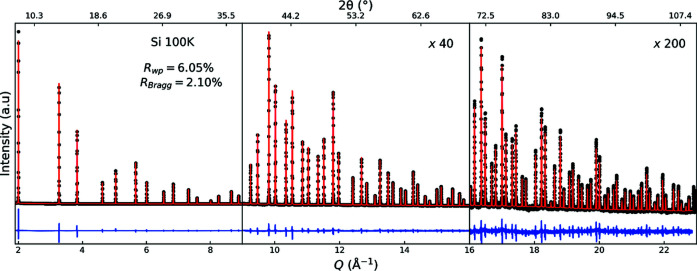
Rietveld analysis results of the total-scattering data of Si at 100 K obtained using OHGI. Black dots, red lines and blue lines show the data points, the calculated pattern and the difference, respectively.

Fig. 1 ▸ shows the results of Rietveld analysis of the total-scattering data of Si collected at 100 K in the *Q* range from 1.9 to 23 Å^−1^. The *R*_wp_ and *R*_Bragg_ values are 6.05% and 2.10%, respectively. The low *R* factors demonstrate that the simple Rietveld model employed sufficiently describes the data, although further improvement possibly could be achieved by accounting for chemical bonding effects via multipole modelling (Svane *et al.*, 2021 ▸).

**Figure 2:**
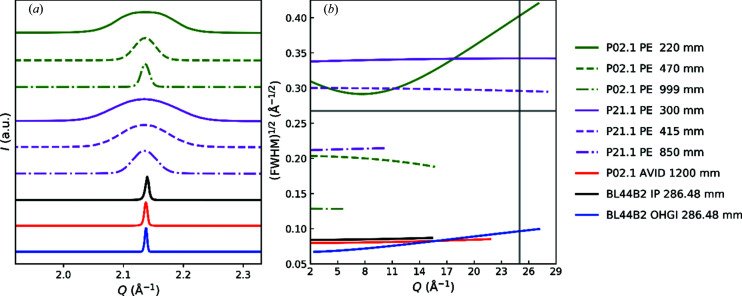
(*a*) Peak shapes of the most intense reflection 110 from LaB_6_ at *Q* ≃ 2.14 Å^−1^ measured at different beamlines. (*b*) The square root of FWHM of the LaB_6_ peak profiles as a function of *Q*, which were computed by the TCH parameters refined by the Le Bail method. The vertical grey line at *Q* = 25 Å^−1^ and the horizontal grey line at (FWHM)^1/2^ = 0.27 Å^−1/2 ^ represent the minimum *Q* range and *Q* resolution, respectively, required for dual-space-quality total-scattering data. Data were collected at three different beamlines: P02.1 and P21.1 at PETRA III, and BL44B2 at SPring-8. PE and the following numbers denote a Perkin–Elemer area detector and the sample-to-detector distances, respectively. Experimental details such as X-ray wavelengths, capillary type and beam dimensions can be found in Table S1 in the Supporting information.

To evaluate the instrumental resolution of OHGI, the LaB_6_ data were compared with other synchrotron beamlines with different detectors dedicated to *in situ* or *operando* measurements. The resolution has also been compared with the IP detector at beamline BL44B2 at SPring-8 and the Aarhus IP detector (AVID) (Wahlberg *et al.*, 2016 ▸; Tolborg *et al.*, 2017 ▸). Fig. 2 ▸(*a*) shows the peak shapes of the most intense reflection and Fig. 2 ▸(*b*) shows the square root of the full widths at half maxima (FWHM) as a function of *Q*, which was calculated from the TCH parameters refined by the Le Bail method. For comparison, the FWHM that were originally computed in 2θ were transformed into the corresponding values in *Q* by using the approximation for sufficiently small values given by Δ*Q* = 4π cos θ/λΔθ. The vertical and horizontal grey lines on Fig. 2 ▸(*b*) show levels of *Q* = 25 Å^−1^ and (FWHM)^1/2^ = 0.27 Å^−1/2^, respectively. Given a constant Gaussian peak-profile function, this value of (FWHM)^1/2^ is where the corresponding PDF is damped down to one percent at *r* = 100 Å. It is thus necessary to collect data with an instrumental resolution below the horizontal line to produce long-range PDFs. The vertical line denotes the minimum *Q* range for producing PDFs with a direct-space resolution of ∼0.125 Å (calculated from Δ*r* ≃ π/*Q*_max_). This resolution is still too low for peak separation in some structures (Qiu *et al.*, 2004 ▸) but serves as a minimum requirement for high-quality PDFs.

The results in Fig. 2 ▸(*b*) clearly indicate that OHGI satisfies the two criteria for long-range high-resolution total-scattering measurements, enabling dual-space analysis.. The Perkin–Elmer (PE) area detectors employed at P02.1 or P21.1 with different sample-to-detector distances do not satisfy the criteria owing to the trade-off relationship between *Q* range and *Q* resolution. Both AVID and the IP detector at BL44B2 have comparable resolutions with OHGI but their *Q* ranges do not satisfy the criteria.

**Figure 3:**
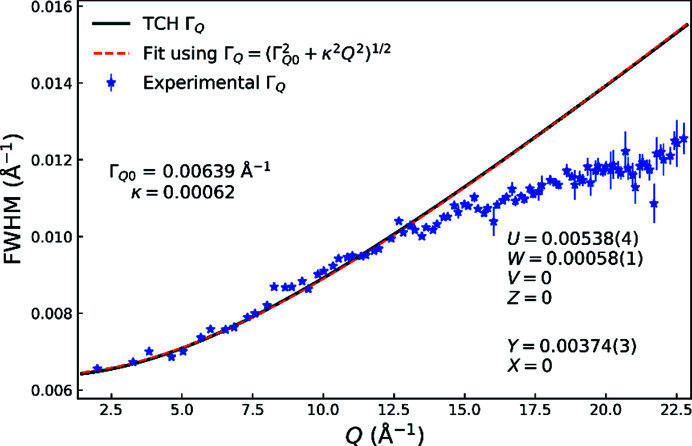
FWHM of Si at 100 K. The blue stars show experimental FWHM from single-peak fitting using a generic pseudo-Voigt function. The black line shows the FWHM function obtained from the refined TCH parameters in Rietveld analysis of the Si 300 K data. The red dashed line shows a least-squares fit to the TCH FHWM using the function 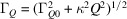. The refined values of Γ_*Q*0_ and κ are shown alongside the refined TCH peak-profile parameters *U*, *V*, *W*, *Z*, *X* and *Y*.

The instrumental *Q* resolution of total-scattering data depends on the divergence and energy resolution of the primary beam, as well as the point (or line) spread function of the detector. The instrumental *Q* range is determined by the energy of the primary beam and the architecture of the detector system. As shown in Fig. 2 ▸(*b*), both have been optimized for OHGI to yield dual-space-quality total-scattering data. In Fig. 3 ▸, the experimental FWHM from single-peak fitting and FWHM as calculated from the refined TCH parameters from the Rietveld analysis of Si at 100 K are shown. A peak-profile function of 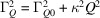 has been fitted to the TCH FWHM, where Γ_*Q*0_ is a constant contribution to the peak width and κ is a linear broadening coefficient. This peak-profile function follows refined TCH FWHM very closely but a deviation from the experimental FWHM is seen at high *Q*. The discrepancy in FWHM is also noticeable in the difference curve at high *Q* in Fig. 1 ▸, where every peak has a higher maximum intensity than the model owing to overestimation of the FWHM. Inspection of the individual fits (see Fig. S1 in the Supporting information) shows that the shape of the generic pseudo-Voigt function used for single-peak fitting gives an adequate description, which means that the discrepancy is solely in the width of the peaks. Many combinations of TCH parameters were tested to improve the Rietveld model at high *Q* but those reported in Fig. 3 ▸ gave the lowest agreement factors owing to the high intensity, and consequently high weight, of the low *Q* diffraction peaks. The refined TCH shape is primarily Gaussian with a Lorentzian mixing parameter between 18.4% and 7.86% for peaks in the lowest and highest reciprocal-space regions, respectively.

Considering the measured intensity of OHGI, the precision and accuracy are significantly influenced by XRNU, as described in Section 1. To correct the data for XRNU, correction factors were obtained with ReLiEf (Kato *et al.*, 2019 ▸; Kato & Shigeta, 2020 ▸) at the incident wavelength and energy threshold that were identical to those used for sample measurement. To investigate for any systematic intensity errors, total-scattering data obtained using the appropriate XRNU correction factors have been examined in terms of ADPs of Si at 100 and 300 K. The ADPs of Si at these temperatures are established from theory and previously reported experiments.

Table 1 ▸ shows the extracted ADPs of Si with reference values (Wahlberg *et al.*, 2016 ▸; Tolborg *et al.*, 2017 ▸; Flensburg & Stewart, 1999 ▸; Sang *et al.*, 2010 ▸). The ADP values at both temperatures from OHGI agree well with those from other experiments even though they are somewhat smaller in all cases, except for AVID #1. This inconsistency can be explained by the coexistence of Bragg scattering and thermal diffuse scattering (TDS). Since the integrated intensity of each Bragg peak at high 2θ angles tends to be overestimated owing to TDS (Willis & Pryor, 1975 ▸), the refined ADP values become an underestimation of the true values if TDS is not accounted for. The fact that this effect is noticeable at 300 K is a testimony to the high precision of weak scattering on the OHGI instrument. The precision is on par with AVID, which has previously served as a benchmark for state-of-the-art PXRD data quality.

## 4. Pair distribution functions   

**Figure 4:**
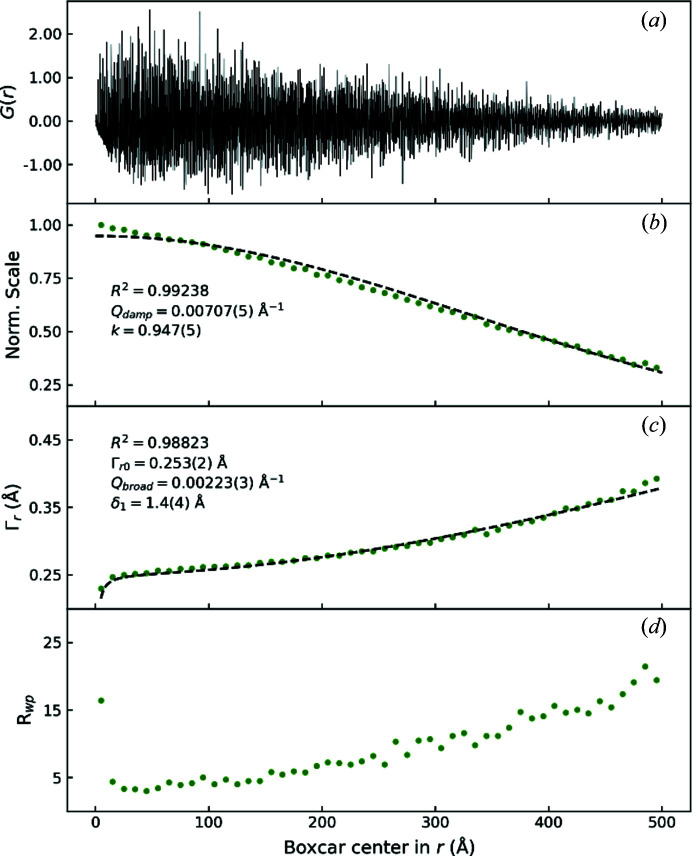
(*a*) The PDF of Si at 300 K with a range of *r* = 500 Å based on the total-scattering data collected with OHGI. (*b*)–(*d*) Boxcar-refinement results of the PDF for normalized scale factors (*b*), the refined FWHM Γ_*r*_ (*c*) and *R*_wp_ values (*d*). The refined values are shown by green dots. The Γ_*r*_ values were calculated using the equation Γ_*r*_ = 2[2 ln(2)]^1/2^(*U*_1_ + *U*_2_)^1/2^, where *U*_1_ and *U*_2_ are the ADPs of the two atoms separated by the interatomic distance *r*. In the case of Si, *U*_1_ = *U*_2_ = *U*_Si_, which leads to 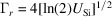. The dashed lines in (*b*) and (*c*) represent least-squares fits with the correction functions for PDF damping and peak broadening given by Q_damp_ and Q_broad_, respectively. *R* ^2^ denotes the correlation coefficient of the fit and *k* in (*b*) is the intercept with the vertical axis.

Fig. 4 ▸(*a*) shows the PDF of Si at 300 K obtained from the total-scattering data collected with OHGI. The long-range PDF clearly demonstrates that interatomic correlations can be observed at least up to *r* = 500 Å thanks to the high *Q* resolution. To examine the characteristics of the long-range PDF, boxcar refinements were carried out. The results are shown in Figs. 4 ▸(*b*)–4 ▸(*d*). When the box was shifted to higher correlations lengths, two conspicuous effects were confirmed. One, shown in Fig. 4 ▸(*b*), is a decrease in PDF peak intensity as a function of *r*, and the other, shown in Fig. 4 ▸(*c*), is a gradual increase in PDF peak width with increasing *r*. In PDF refinements with *PDFgui* (Farrow *et al.*, 2009 ▸), these two effects, *i.e.* the *r*-dependent damping and peak broadening, can be described by the correction parameters *Q*_damp_ and *Q*_broad_, respectively.

The *Q*_damp_ parameter models the width of a Gaussian envelope function. This description is formulated by assuming constant Gaussian peaks for the reciprocal-space peak-profile function. According to the Fourier convolution theorem, the PDF should consequently be multiplied by the Fourier transformation of a constant Gaussian peak profile, which is also a Gaussian. For convenience, the envelope function is typically expressed such that *Q*_damp_ = Γ_*Q*0_, where Γ_*Q*0_ is the FWHM of the peak profiles. As seen in Fig. 4 ▸(*b*), the refined scale factors were successfully fitted by a Gaussian envelope with the exception of those at low *r*. The misfit may be attributed to the Lorentzian component of the peak profiles, which is not taken into account in the *Q*_damp_ description.

**Figure 5:**
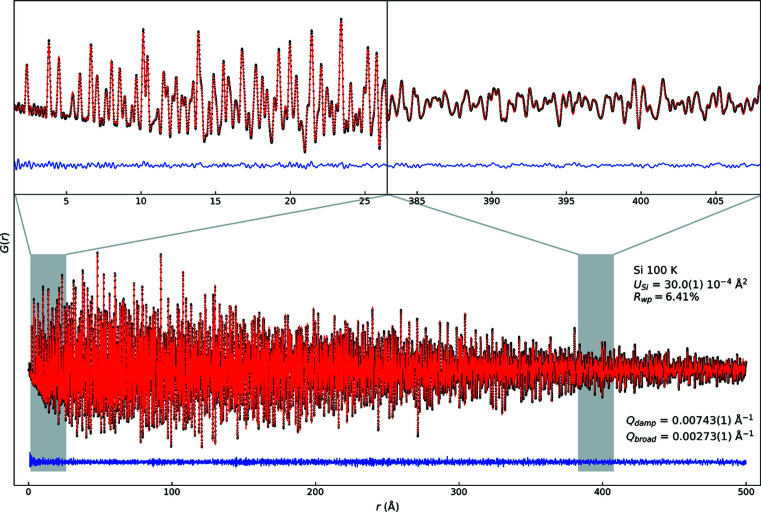
PDF analysis results of the Si data at 100 K in a range up to *r* = 500 Å. Data points, the calculated model and their differences are shown by black dots, a red line and a blue line, respectively.

The *Q*_broad_ parameter models the PDF peak broadening caused by the broadening of the reciprocal peak profiles (Thorpe *et al.*, 2002 ▸). This parameter becomes especially significant for refinements with a wide range in *r*. In the derivation of *Q*_broad_, the *Q*-dependent broadening is assumed to be in accordance with the form 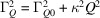, which reproduced the FHWM function in the Rietveld refinement on the OHGI data (see Fig. 3 ▸). Consequently, the PDF peak broadening can be expressed by the following function, as implemented in *PDFgui* (Farrow *et al.*, 2009 ▸),
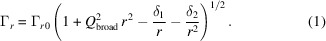



Here, Γ_*r*_ is the total PDF peak width and Γ_*r*0_ is the constant contribution. The two terms δ_1_ and δ_2_ are parameters for correlated atomic motion at higher and lower temperatures than the Debye temperature, respectively (Jeong *et al.*, 2003 ▸). Although the Debye temperature of Si is much higher than room temperature, δ_1_ rather than δ_2_ was employed as a refinement parameter for robustness. In Fig. 4 ▸(*c*), it is shown that this description gives an adequate fit with the refined FWHM from the boxcar refinement. Fig. 5 ▸ shows the whole-range refinement of the 100 K Si PDF up to *r* = 500 Å. The refined model yielded a low *R*_wp_ factor, especially considering the high number of data points. The refined ADP value is also reasonable and close to that found in the PXRD analysis, see Table 2 ▸. In addition, the *Q*_damp_ parameter was one order of magnitude smaller than that at typical PDF beamlines and was comparable with that at high-resolution powder-diffraction beamlines (Saleta *et al.*, 2017 ▸). Once again, these results clearly demonstrate that OHGI can yield highly reliable and well resolved total-scattering data.

Table 2 ▸ lists the structural parameters and *R*_wp_ factors obtained from reciprocal- and direct-space refinements of the Si data at 100 and 300 K. The lattice parameters at both temperatures from reciprocal space agreed with those from direct space on a scale of 10^−4^ Å. The ADP at 100 K from reciprocal space was consistent with that from direct space within the estimated standard deviation. In contrast, the ADP at 300 K from reciprocal space was significantly smaller than that from direct space. As discussed in Section 3, correlated atomic motion results in TDS in and around the Bragg peaks, which causes an artificial decrease in the ADP in reciprocal space when not accounted for. In direct space, the addition of the δ parameters to the model makes it possible to separate the effects of TDS from the ADP.

The overall agreement between the structural parameters for Si in reciprocal and direct space demonstrates that OHGI can provide a measurement basis for single-shot dual-space structural analysis. In the present study, dual-space analysis using a single data set was performed separately. A concurrent dual-space analysis would need to overcome the following challenges for treatment of reciprocal- and direct-space data on an equal basis: (i) how is the agreement of the model in individual spaces weighted, (ii) how is the structural PDF model of polyatomic specimens calculated (Neder & Proffen, 2020 ▸), (iii) how are various structural effects causing diffuse scattering (such as TDS) handled in both spaces simultaneously and (iv) how do the pseudo-Voigt peak profiles with non-negligible Lorentzian components affect the PDF.

## 5. Conclusions   

In conclusion, we found that synchotron total-scattering data obtained through OHGI at BL44B2 at SPring-8 were of unprecedented quality for both accurate PXRD and PDF analysis. Both the lattice parameters and ADPs of Si at 100 and 300 K in direct space were found to be consistent with those in reciprocal space. The correction parameters *Q*_damp_ and *Q*_broad_ were found to adequately describe the effects of the primarily Gaussian reciprocal-space peak profiles on the long-range PDFs (*r* = 500 Å). These results clearly demonstrate that the data quality of single-shot measurements from OHGI is applicable to dual-space analysis and can bridge the gap between the analysis of the average and local structures of crystalline materials.

## Supplementary Material

Supporting information. DOI: 10.1107/S2052252521001664/ro5024sup1.pdf

## Figures and Tables

**Table 1:** Comparisons of the ADPs of Si between the present study and previous reports All the values in the table are shown in units of 10^−4^ Å^2^, unless stated otherwise. INS and CBED stand for inelastic neutron scattering and convergent-beam electron diffraction, respectively.

Temperature (K)	PXRD from OHGI (Present study)	PXRD from AVID #1 (Wahlberg *et al.*, 2016 ▸)	PXRD from AVID #2 (Tolborg *et al.*, 2017 ▸ *)*	INS (Flensburg & Stewart, 1999 ▸)	CBED (Sang *et al.*, 2010 ▸)
100	29.7 (2)	31.81†	33.70†	—	34 (2)
300	57.7 (3)	56.20†	61.03†	59.41 (21)	61 (1)

† Standard deviations were not reported.

**Table 2:** Structural parameters from the reciprocal- and direct-space refinements of Si at 100 and 300 K

Parameters	Reciprocal space	Direct space
For 100 K		
*a* (Å)	5.430014 (3)	5.430057 (1)
*U*_Si_ (10^−4^ Å^2^)	29.7 (3)	30.0 (1)
*R*_wp_ (%)	6.05	6.41
For 300 K		
*a* (Å)	5.431302 (3)	5.431324 (1)
*U*_Si_ (10^−4^ Å^2^)	57.7 (3)	59.5 (1)
*R*_wp_ (%)	6.30	6.06
