# Supplementary material for: Synchrotron total-scattering data applicable to dual-space structural analysis
Source: IUCrJ. 2021 Mar 6;8(Pt 3):387–94. doi: 10.1107/S2052252521001664 (PMC8086158; doi:10.1107/S2052252521001664)
Supplement: Supplementary file 1 [file m-08-00387-sup1.pdf]

IUCrJ

Volume 8 (2021)

Supporting information for article:

Synchrotron total-scattering data applicable to dual-space structural analysis

Jonas Beyer, Kenichi Kato and Bo Brummerstedt Iversen

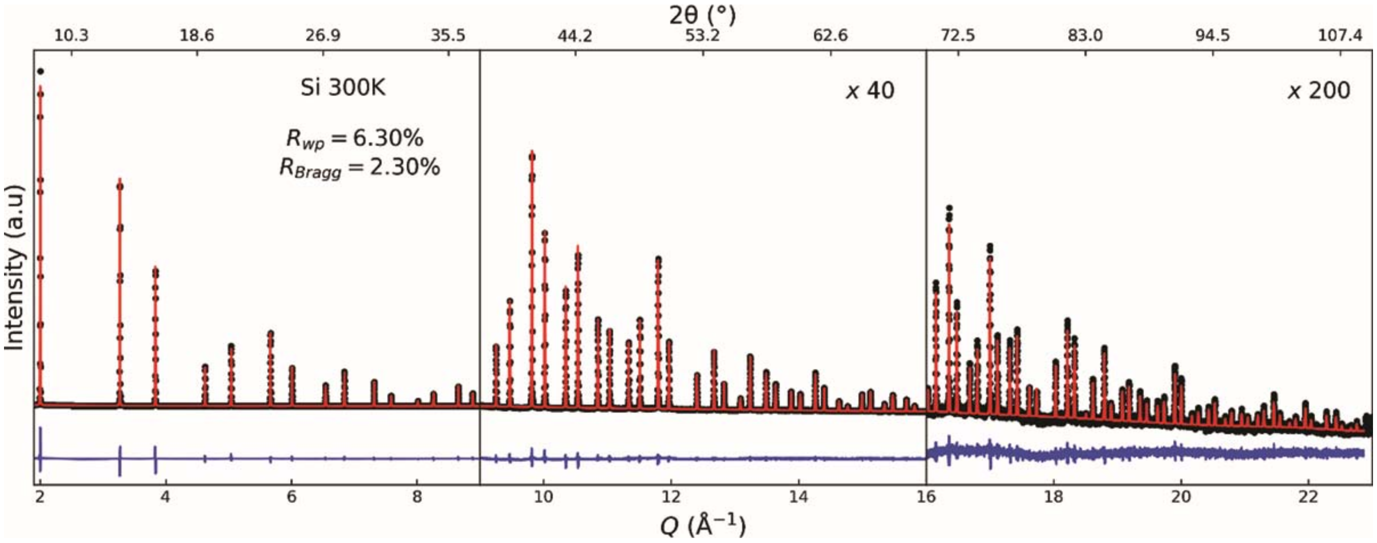

Figure S1 Rietveld analysis results of the total scattering data of Si at 300 K obtained through OHGI. Black dots, red lines, and blue lines show data points, calculated model, and their differences, respectively.

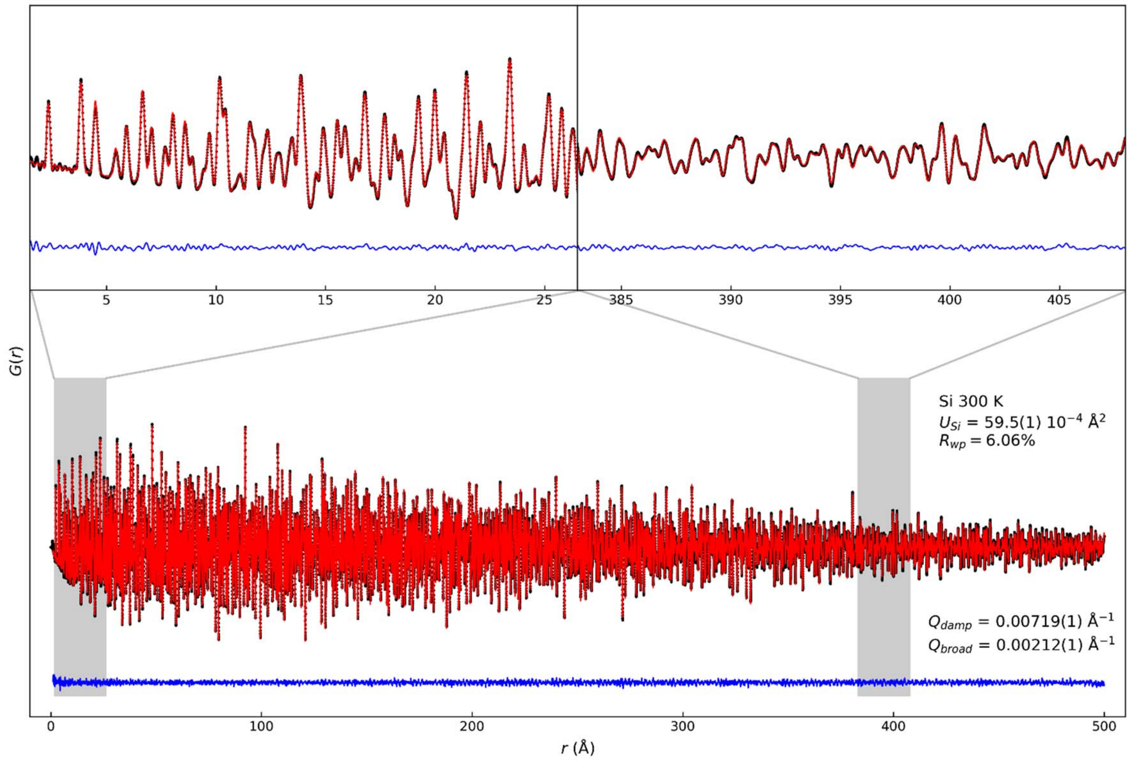

Figure S2 PDF analysis results of the Si data at 300 K in a range up to r = 500 Å. Black dots, red lines, and blue lines show data points, calculated model, and their differences, respectively.

Table S1 Experimental details for LaB_6 data

Facility	Beamline and detector	Sample-to-detector distance (mm)	Experiment Date	Energy (keV)	Capillary material and diameter (mm)	Beam size (horizontal x vertical) (mm^2)
PETRA III	P21.1 PE	300	Sep. '19	100	Kapton 1.0	0.8 x 0.8
	P21.1 PE	415	Nov. '19	100	Kapton 1.0	1.2 x 1.2
	P21.1 PE	850	Nov. '19	100	Kapton 1.0	1.2 x 1.2
	P02.1 PE	220	Dec. '18	60	Kapton 1.0	1 x 1
	P02.1 PE	470	Dec. '18	60	Kapton 1.0	1 x 1
	P02.1 PE	999	Nov '16	60	Kapton 1.0	1 x 1
	P02.1 AVID	1200	April '18	60	Glass 0.2	0.3 x 0.3
SPring-8	BL44B2 Image-plate	286.48	Dec. '16	24.80	Glass 0.1	3.0 x 0.5
	BL44B2 OHGI	286.48	Oct. '18	27.46(6)	Glass 0.1	3.0 x 0.5

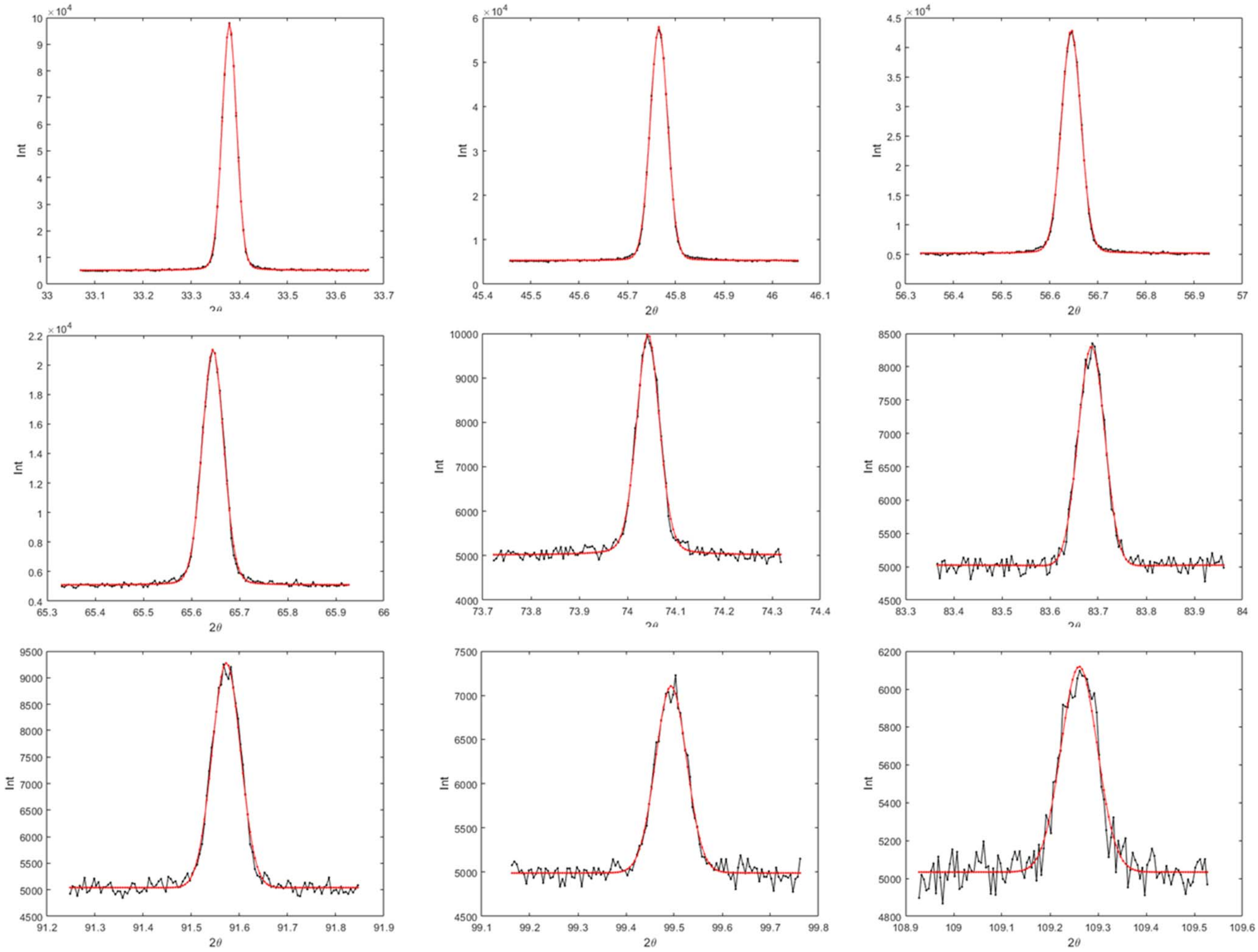

Figure S3 Single peak fits of diffraction peaks from Si 100K.
